# Relationship between homocysteine, vitamin B9, vitamin B12 levels methylenetetrahydrofolate reductase (C677T, A1298C) polymorphisms, and cryptogenic stroke in Tunisian adults´ patients: a case-control study

**DOI:** 10.11604/pamj.2024.48.111.41629

**Published:** 2024-07-17

**Authors:** Lamia Mbarek, Salma Sakka, Aida Elleuch, Ayadi Mohsen, Sawsan Daoud, Khadija Moalla, Nadia Bouattour, Mariem Dammak, Fatma Ayadi, Chokri Mhiri

**Affiliations:** 1 Clinical Investigation Center, Habib Bourguiba University Hospital of Sfax, Sfax, Tunisia,; 2 Department of Neurology, Laboratory of Neurogenetics, Parkinson´s Disease and Cerebrovascular Disease (LR-12-SP-19), Habib Bourguiba University Hospital, University of Sfax, Sfax, Tunisia,; 3 Biochemistry Laboratory, Habib Bourguiba University Hospital of Sfax, Sfax, Tunisia,; 4 Research laboratory, “Molecular Basis of Human Pathology”, LR19ES13, University of Sfax, Sfax, Tunisia

**Keywords:** Homocysteine, vitamin B9, vitamin B12, MTHFR C677T, MTHFR A1298C

## Abstract

**Introduction:**

the relationship between elevated plasma homocysteine (Hcy) and stroke has been established, but this association remains indistinct in cryptogenic stroke in adults. Our aim is to investigate the association between homocysteine, vitamins B9 and B12, and cryptogenic stroke. Furthermore, to determine the correlation between methylenetetrahydrofolate reductase (MTHFR) polymorphism and biochemical levels in plasma.

**Methods:**

we conducted a retrospective case-control study including 100 cryptogenic stroke patients aged 18-50 years and 100 participants with age-and-sex-matched healthy controls. Clinical, radiological, and outcome data from cerebral venous thrombosis (CVT) patients were recorded. Homocysteine, vitamin B9, and vitamin B12 were analyzed. Deoxyribonucleic acids (DNAs) from both groups were tested. MTHFR C677T mutation was assessed by restriction fragment length polymorphism (PCR). All analyses were performed using Statistical Package for the Social Sciences (SPSSV.20) software. Multivariable logistic regression analysis was performed to identify factors associated with stroke risk and clinical outcomes.

**Results:**

a total of 200 cases were included in this study, 50% (n=100) patients with cryptogenic stroke (mean age of 40.49 ± 6.2 years, sex-ratio= 1.5) and 50% (n=100) healthy cases (mean age of 39.09 ± 5.8 years, sex-ratio= 1.5). The elevated plasma level of Hcy and vitamin B9 levels deficiency increase the risk of cryptogenic stroke occurrence (aOR: 2.5; 95% (0.71-5.25), P=0.01), (aOR: 3.1; 95% (1.6-9.6), p=0.02 respectively). Additionally, vitamin B9 deficiency was significantly associated with elevated Hcy levels (4.57 ± 3.59; p=0.001). Genetic analysis revealed a significant association between homozygous TT genotype of the MTHFR C677T polymorphism, elevated Hcy levels (20.4 ± 7.07; p=0.001) and vitamin B9 deficiency (4.9±3.9; p=0.001). Furthermore, the combined CT/AC genotype was associated with elevated Hcy level (21.6 ± 9.6; p=0.001) and vitamin B9 deficiency (2.9 ± 1.0; p=0.04).

**Conclusion:**

the presence of homozygote MTHFR C677T or dual heterozygous MTHFR C66T and A1298C, which leads to elevated Hcy and deficiency of vitamin B9 plasma levels, is correlated with an increased risk of cryptogenic stroke occurrence among adult Tunisian patients.

## Introduction

Cryptogenic stroke is a complex condition characterized by an ischemic event for which no definite cause is found despite thorough diagnostic examination. The definition of cryptogenic stroke varies, often drawing from the classifications in the trial of ORG 10172 in Acute Stroke Treatment (TOAST) [1]. According to this framework, cryptogenic strokes are those that cannot be attributed to specific sources such as cardioembolism, large artery atherosclerosis, small artery disease, or any other uncommon known cause, even after standard assessments. This category may include strokes with incomplete evaluations or multiple potential causes. It is estimated that approximately 25-40% of all ischemic strokes fall into the cryptogenic category [2]. Although advances in diagnostic techniques have reduced the number of ischemic strokes of undetermined origin over time, cryptogenic stroke remains a significant challenge for neurologists, as treatment decisions heavily rely on understanding the underlying stroke mechanisms. Several studies have identified an association between elevated Hcy levels and the risk of all subtypes of stroke [3]. Hcy is a demethylated by-product of methionine, and as such, is regarded as a toxic amino acid. It is typically eliminated by methionine synthase in a remethylation process that utilizes methylcobalamin as a co-factor and 5-methyl tetrahydrofolate as a methyl donor. In the brain, the catabolism of Hcy is significantly dependent on its remethylation to methionine, which is accomplished through the use of methylcobalamin and vitamin B9 [4]. It has been demonstrated that B vitamins namely vitamin B6, vitamin B12, and vitamin B9 are essential co-factors during the catabolism process of Hcy. Furthermore, an effective Hcy re-methylation requires the enzyme methylenetetrahydrofolate reductase (MTHFR). The MTHFR is a key enzyme that irreversibly catalyzes the conversion of 5,10-MTHFR to 5-MTHF and plays a vital role in governing the distribution of folic acid throughout the entire metabolic pathway [5].

Genetic polymorphisms in the MTHFR gene can impact enzyme activity. In vivo, the C677T and A1298C polymorphisms can reduce up to 75% of enzyme activity [6]. MTHFR polymorphisms are supposed to be related to coronary diseases, venous thrombotic events, epilepsy, different obstetrical complications, and ischemic stroke in adults [7-9]. The relationship between Hcy, vitamin B9, and vitamin B12, as well as MTHFR polymorphisms, and the occurrence of ischemic and hemorrhagic strokes, has been well-established. This understanding could pave the way for the development of innovative treatment strategies aimed at stroke prevention. However, their association with cryptogenic stroke remains unclear.

This study aims to investigate the association between Hcy, vitamin B9, vitamin B12, MTHFR polymorphisms, and the occurrence of cryptogenic stroke in a case-control study involving both cryptogenic stroke patients and healthy adults. Secondly, we seek to demonstrate the relationship between elevated Hcy levels, and the vitamins B9, and B12 levels, MTHFR polymorphisms, and neurological severity scores.

## Methods

**Study design:** this is a case-control study, to assess the association between Hcy, vitamin B9, vitamin B12, MTHFR polymorphisms, and the occurrence of cryptogenic stroke.

**Study setting:** this study was conducted in the Department of Neurology, at Habib Bourguiba University Hospital of Sfax, Tunisia, between January 2018 and September 2020. We performed a biochemical workup and genetic analysis. This data was collected after participant's consent.

**Study population:** we include 200 cases aged between 18 to 50 years, among them 100 cryptogenic stroke patients. The diagnosis of cryptogenic stroke was based on the clinical manifestations, brain computed tomography (CT), and/or magnetic resonance imaging (MRI). The etiology subtype of stroke was classified based on TOAST classification; cryptogenic patients were referred to undetermined causes of stroke. Patients with hyperthyroidism, systemic diseases, brain tumors other oncologic pathologies, and psychiatric diseases such as psychosis were excluded from our study.

**Data collection:** each subject in the study underwent a complete neurological and clinical examination, as well as complementary biological and radiological tests. Demographic characteristics include age, gender, obesity, and lifestyle including current smoking, and severe alcohol drinking. As well, clinical risk factors such as hypertension, diabetes, and dyslipidemia were collected using a pre-established form.

The control group included 100 healthy cases without a personal history of thrombosis matched in gender and age with the patient's group.

**Laboratory analysis:** blood was drawn from fasting patients and collected in tubes containing ethylenediaminetetraacetic acid (EDTA). Plasma Hcy, vitamin B9, and vitamin B12 specimens were placed on ice and transported to the laboratory within 30 minutes of collection. The blood was centrifuged between 1 and 2 hours after venipuncture. Plasma was frozen at 22°C until analysis. Total Hcy, vitamin B9, and B12 concentrations were determined by using an automatic analyzer based on the fluorescence polarization technique (FPIA) on Cobas 6000 from Roche. A threshold of 12 μmol/l was adopted to define elevated homocysteine. The standard vitamin B9 and B12 were between 4.5 - 20 ng/ml and 197 - 771 pg/ml, respectively.

**Genotyping analysis:** genomic DNA was extracted from peripheral blood leukocytes according to the phenol-chloroform protocol. The MTHFR gene polymorphism was detected using the restriction fragment length polymorphism (PCR-RFLP) method. The PCR products were digested with *HinfI* and *MboII* restriction enzymes respectively.

**Statistical analysis:** all analyses related to the case-control study were performed using the Statistical Package for the Social Sciences V.20 (IBM (formerly SPSS, Inc.)). Data on quantitative characteristics are expressed as means ± SD. Differences between cases and controls were evaluated by using the chi-square test for qualitative variables and the student t-test for quantitative variables. Multivariable logistic regression analysis was performed to identify factors associated with stroke risk and clinical outcomes. In addition, the odds ratio (OR), adjusted OR (aOR), and 95% confidence intervals respectively were calculated. Probability values, P < 0.05, were considered statistically significant.

**Ethical consideration:** The study protocol was approved by our ethics committee.

**Informed consent:** all subjects of the study participated after they had given their fully informed consent.

## Results

**General characteristics of the study population:** this case-control study included two age- and sex-matched groups: 100 patients with cryptogenic stroke, with a mean age of 40.49 ± 6.2 years and a sex ratio of 1.5; and 100 healthy controls, with a mean age of 39.09 ± 5.8 years and a sex ratio of 1.5. Hypertension, diabetes metillus, hypercholesterolemia, oral contraceptives and smoking were significantly associated with androgen insensitivity syndrome (AIS) occurrence. Whereas, no association between AIS occurrence and alcohol intake in our study. Clinical data are summarized in Table 1. The data obtained regarding the plasma levels of biochemical mediators revealed that high mean plasma Hcy and low vitamin B9 levels were prominently associated with a heightened AIS risk in young adults. The mean Hcy level increased the risk of AIS by 2.9 times (aOR:3.1; 95% (1.6-9.6), P=0.02). This risk was enhanced in the presence of vitamin B9 plasma level deficiency (aOR:3.1; 95% (1.6-9.6), P=0.02) while it was not influenced by the low vitamin B12 plasma level.

**Table 1:** sample characteristics for the case-control study

Risk factors	Patients N (%)	Control N (%)	OR	95% CI)	P	aOR	(95% CI)	P
Age (years)	40.49 ± 6.2	39.09 ± 5.8	1.10	0.62-1.3	0.55	0.7	0.25-2.26	0.56
Gender (male)	60 (60)	60 (60)	1.00	0.71-1.40	0.99	0.96	0.5-1.5	0.7
Smoking	51(51%)	24 (24%)	3.29	1.8-6.02	<0.001	3.1	1.2-5.4	0.03
Hypertension	32 (32%)	9 (9%)	4.75	7.21-10.62	<0.001	2.006	1.18-3.4	<0.001
Diabetes	25 (25%)	5 (5%)	6.33	2.31-17.33	<0.001	6.43	1.01-9.44	<0.001
Dyslipidemia	25 (25%)	4 (4%)	3.9	1.23-12.32	0.01	2.8	1.7-9.3	0.01
Oral contraception	5 (5%)	5 (5%)	3.86	1.43-10.39	0.004	1.5	0.7-3.2	0.5
Hcy	17.37 ± 8.8 (74%)	13.47 ± 4.54 (58%)	2.95	1.8-4.83	0.025	2.5	0.71- 5.25	0.01
Vitamin B9	3.55 ± 2.38 (79%)	8.71 ± 3.55 (6%)	3.94	2.12-7.31	<0.001	3.1	0.6-9.6	0.02
Vitamin 12	372 ± 238 (14%)	351 ± 124 (9%)	2.89	0.08-8.34	0.14	2.34	1.5-7.3	0.5

Hcy: homocystein; N: number of cases; OR: unadjusted odds ratio; aOR: adjusted odds ratio for age, gender, smoking, hypertension, diabetes, and oral contraception; P: p-values at alpha 0.05

**Relationship of vitamin deficiencies on homocystein levels:** Table 2 shows the effect of vitamin deficiencies on Hcy levels. We found that vitamin B9 deficiency (4.57 ± 3.59 ng/ml) was significantly associated with increased Hcy levels (p=0.001). However, the plasma Hcy level is not influenced by vitamin B12 deficiency (p= 0.60). In addition, we analyzed the effect of a combined deficiency of vitamin B12 and vitamin B9 on the level of Hcy. This multivariate analysis showed that combined vitamin B9 and vitamin B12 deficiency was not significantly associated with elevated plasma Hcy (OR=1.008; 95% CI (0.09-11.316); p=0.995).

**Table 2:** relationship between homocysteine group and vitamins B9 and B12 plasma levels in cryptogenic patients

Homocysteine (μmol/L)	(<12 μmol/L) (26%)	(>12μmol/L) (74%)	P
Vitamine B12 (pg/mL)	384,17 ± 143.81	350.43 ± 208.52	0.204
Vitamine B9 (ng/mL)	7.23 ± 4.22	4.57 ± 3.59	0.011

P: p-values at alpha 0.05

**Relationship between isolated genetic polymorphisms and plasma homocysteine, vitamin B9, and vitamin B12 levels:** Table 3 exhibits the relationship between MTHFR (C677T, A1298C) and the plasma levels of Hcy, vitamin B9, and vitamin B12. A significant association between the homozygous TT genotype of the MTHFR 677 polymorphism and elevated Hcy levels (20.4 ± 7.07 µmol/l; p < 0.001), as well as a vitamin B9 deficiency (4.93 ± 3.9 ng/ml; p < 0.001), was disclosed. No alteration was observed with the other MTHFR 677CT, 1298AC, and 1298 CC polymorphisms.

**Table 3:** impact of methylenetetrahydrofolate reductase (MTHFR) polymorphisms on Hcy, vitamin B9, and vitamin B12 levels

MTHFR	Homocysteine(µmol/L)		Vitamin B9 (ng/mL)		Vitamin B12 (pg/mL)	
677CC	10.1 ± 4.07	-	3.23 ± 1.53	p=0.47	358.8 ± 192.7	p=0.81
677CT	14.1 ± 8.29	p=0.42	3.15 ± 1.54	p=0.23	339.1 ± 191.7	p=0.42
677TT	20.4 ± 7.07	p=0.001	4.93 ± 3.9	p<0.001	361.6 ± 166.8	p=0.94
1298AA	11.4 ± 6.4	p=0.36	3.63 ± 2.47	p=0.27	388.2 ± 301	p=0.36
1298AC	15.8 ± 8.5	p=0.96	2.98 ± 1.21	p=0.1	364.3 ± 301.1	p=0.9
1298CC	14.2 ± 5.3	p=0.67	3.91 ± 2.52	p=0.39	376 ± 145.8	p=0.2

MTHFR: methylenetetrahydrofolate reductase

**Relationship between combined genetic polymorphisms and plasma homocysteine, vitamin B9, and vitamin B12 levels:** we assessed the potential impact of the combined MTHFR C677T and A1298C polymorphisms. Table 4 presents the relationship between the combined genetic polymorphisms and the plasma levels of Hcy, vitamin B9, and vitamin B12. The combined polymorphism CT/AC was associated with an elevated plasma Hcy level (21.61 ± 9.66 µmol/l; p < 0.001) and a vitamin B9 deficiency (2.94 ± 1.02 ng/ml; p=0.04). The combination of both TT/AC polymorphisms was associated with higher Hcy levels (24 ± 5.2 µmol/l; p=0.02).

**Table 4:** impact of combined methylenetetrahydrofolate reductase (MTHFR) polymorphisms and Hcy, vitamin B9 and vitamin B12 levels

677/1298	Hcy(µmol/L)	P	Vitamin B9 (ng/mL)	P	Vitamin B12 (pg/mL)	P
CT/AC	21.61± 9.66	0.002*	2.94 ± 1.02	0.004*	425±389.8	0.89
CT/CC	15.6 ± 5.4	0.4	4.66 ± 3.9	0.4	290±147	0.6
TT/AC	24 ± 5.2	0.02*	3.75±1.7	0.1	218±180	0.8

Hcy: Homocystein level; P-values at alpha 0.05

## Discussion

The results of this case-control study involving 100 adult patients with cryptogenic stroke and 100 healthy individuals unveiled several important findings. Firstly, it was observed that the risk of cryptogenic stroke is heightened in individuals with elevated levels of Hcy and a deficiency in vitamin B9. Furthermore, the study revealed that elevated levels of Hcy and vitamin B9 deficiency were linked to individuals with homozygote MTHFR and those with a combined heterozygote C677T and A1298C. These findings emphasize the significance of monitoring Hcy levels and vitamin B9 status in individuals at risk for cryptogenic stroke.

Cryptogenic stroke is a heterogeneous entity defined as an ischemic stroke for which no probable cause is identified despite thorough diagnostic evaluation [10]. Unlike older patients, adults (<50 years old) with ischemic stroke exhibit a lower incidence of traditional atherosclerosis risk factors, including dyslipidemia, hypertension, diabetes mellitus, and atrial fibrillation, and are less likely to have concomitant coronary or peripheral artery diseases. This ambiguity in etiology persists even with extensive evaluation, with cryptogenic strokes accounting for 30-40% of ischemic strokes in young individuals. The impact of this enigmatic condition extends significantly to adult patients, affecting not only their physical health but also exerting a detrimental influence on their families and the broader societal landscape [11].

Previous studies have highlighted a potential association between elevated levels of Hcy and deficiencies in vitamins B9 and B12 with an increased risk of thromboembolic venous and arterial thrombosis [12,13]. However, the precise relationship with cryptogenic stroke remains unclear.

In our initial study, we sought to investigate the relationship between plasma levels of Hcy, vitamin B9, and B12, and the occurrence of cryptogenic stroke. Our findings indicated a significant association between elevated plasma Hcy levels and vitamin B9 deficiency, which was associated with an increased risk of cryptogenic stroke. Our findings align with previous research indicating that elevated plasma Hcy levels can result in a reduction in nitric oxide bioavailability, leading to endothelial dysfunction, vascular damage, and an elevated risk of atherosclerosis, hypoxic events, lipid peroxidation, and subsequent ischemic attacks [14,15].

Additionally, deficiencies in vitamins B9 and B12 have been associated with hematological and neurological symptoms [16]. Our results indicated that a deficiency in vitamin B9, but not B12, was associated with an increased risk of cryptogenic stroke and elevated Hcy plasma levels. Similar to a previous study, considering that vitamin B9 deficiency as the most frequent cause of hyperhomocysteinemia and stroke [17-19]. Conversely, previous studies have suggested a positive correlation between vitamin B12 and ischemic stroke in older or general patient populations. This discrepancy may be attributed to variations in the age range of the study participants [20,21].

In the second part of this study, we investigate the interactive effect of MTHFR polymorphisms on plasma levels of Hcy, vitamin B9, and vitamin B12. Our results demonstrated that the TT genotype of the MTHFR 677 polymorphism, as well as double heterozygous variations of both MTHFR polymorphisms (C677T/A1298C), were correlated with elevated Hcy and vitamin B9 deficiency. Similarly a recent meta-analysis also demonstrated that the MTHFR TT genotype was associated with increased plasma Hcy and decreased serum vitamin B9 [22]. However, no correlation between MTHFR polymorphisms and vitamin B12 was noted in our study, in contrast to the Jordanian study [23], which suggests a significant association was found between the homozygous MTHFR C677T variant and vitamin B12 deficiency in the Jordanian population (OR:1.684 (1.116-2.542), p:0.017). Moreover, Zittan *et al*. [24] reported that a higher frequency of TT polymorphisms (69%) was associated with vitamin B12 deficiency. The C677T polymorphism is located in exon 4 and leads to an alanine-to-valine amino acid substitution at the corresponding codon, 222. This polymorphism is associated with a reduction in enzyme activity in individuals with the TT genotype, with a reduction of up to 70%, and in those with the CT genotype, with a reduction of up to 35%. The second MTHFR gene polymorphism, A1298C, is located in exon 7, at a distance of 2.1 kb from the MTHFR 677 T position. It results in the substitution of glutamate with alanine at codon 429, which is a relatively minor change in the protein sequence. The latter variant is associated with reduced enzyme activity of approximately 33% [6].

In clinical practice, the efficacy of treatment for reducing Hcy remains a subject of debate. Some studies have indicated that folic acid and/or vitamin B supplementation could serve as an effective means for the prevention of atherosclerotic disease, ischemic stroke, and the enhancement of endothelial function in patients with coronary artery disease [25]. A recent series of randomized clinical trials demonstrated that 5-methyltetrahydrofolate (5-MTHF) exhibits greater efficacy in vivo compared to folic acid [26]. Nevertheless, recent research has demonstrated that while folic acid supplementation maintains adequate vitamin B9 levels in individuals with normal methylenetetrahydrofolate reductase (MTHFR) activity, cells with low MTHFR activity require 5-methyltetrahydrofolate (5-Me-THF) in order to overcome metabolic deficiencies caused by polymorphisms in their MTHFR genes. In addition to folic acid and vitamin B12 therapy, recent research suggests that riboflavin (vitamin B2) supplementation may result in reduced homocysteine concentrations in individuals who are heterozygous for the methylenetetrahydrofolate reductase (MTHFR) gene polymorphism (TT genotype) [27].

The significance of this study lies in the correlation between elevated homocysteine levels and the risk of stroke. Moreover, the study demonstrated the inadequacy of screening for MTHFR polymorphisms in determining the origin of homocysteine elevation and vitamin B9 deficiency. This allows for the selection of an optimal therapeutic strategy. While our study has significant clinical implications, it should be noted that it is limited by the relatively small number of patients included. In future research, it would be beneficial to recruit a larger population. Secondly, further investigation of other polymorphisms with Hcy metabolism is warranted.

## Conclusion

The elevated Hcy and deficiency vitamin B9 plasma levels caused by the presence of homozygote MTHFR C677T or dual heterozygous MTHFR C66T and A1298C were associated with increased risk of cryptogenic stroke occurrence in adult Tunisian patients.

### What is known about this topic

- *Cryptogenic stroke is a heterogeneous disease;*
- *Elevated homocysteine plasma level was associated with increased risk of stroke;*
- *Methylenetetrahydrofolate reductase (MTHFR) is one of the genetic risk factors that has been associated with arterial ischemic stroke*.

### What this study adds

- *Elevated homocysteine and vitamin B9 deficiency were significantly associated with an increase in cryptogenic stroke in adults;*
- *Vitamin B9 deficiency is correlated with elevated homocysteine plasma levels;*
- *The homozygous variant of the MTHFR677 gene and combined heterozygous variant (CT/AC) of MTHFR polymorphisms were correlated with elevated homocysteine and decreased vitamin B9 levels*.
